# “It Helped Me Understand What I Was Walking into:” Youth and Caregiver Acceptability and Satisfaction with a Psychological Perioperative Pain Management Intervention

**DOI:** 10.3390/healthcare13131527

**Published:** 2025-06-26

**Authors:** Nicole E. MacKenzie, Remsha Rana, Lisa Isaac, Jennifer Tyrrell, Danielle Ruskin

**Affiliations:** 1 Department of Psychology, The Hospital for Sick Children, Toronto, ON M5G 1E8, Canada; 2 Department of Psychology and Neuroscience, Dalhousie University, Halifax, NS B3H 4R2, Canada; 3 Department of Anesthesia and Pain Medicine, Hospital for Sick Children, Toronto, ON M5G 1E8, Canada; 4 Department of Anesthesiology and Pain Medicine, Temerty Faculty of Medicine, University of Toronto, Toronto, ON M5S 1A1, Canada; 5 Lawrence Bloomberg Faculty of Nursing, University of Toronto, Toronto, ON M5S 1A1, Canada; 6 Department of Psychology, York University, Toronto, ON M3J 1P3, Canada

**Keywords:** chronic post-surgical pain, psychological intervention, youth, caregivers

## Abstract

**Background:** Chronic postsurgical pain (CPSP) occurs in approximately one in five children who undergo surgery. Youth with anxiety and depressive symptoms are at greater risk of developing CPSP. Psychological interventions hold promise to prevent CPSP; however, existing psychological interventions are often brief and offered exclusively pre-surgically. **Objective:** The Perioperative Pain Program (PPP) was designed to address psychological risk factors for CPSP. This study aimed to explore acceptability and satisfaction with the PPP, from the perspective of youth and caregivers. **Method:** In this mixed-methods study, 24 youth and caregivers completed a satisfaction questionnaire, and six dyads participated in semi-structured interviews. Quantitative data was analyzed using descriptive statistics. Qualitative data was analyzed using inductive content analysis. **Results:** The qualitative analysis generated four categories: (1) developing support and a sense of preparedness for surgery; (2) connection matters between families and the team; (3) personal characteristics may impact intervention use; (4) the need for adaptable content and delivery. Quantitative data indicated overall satisfaction and acceptability of the intervention. **Conclusions:** Psychological interventions that facilitate interpersonal connections in a timely manner may be key to facilitating more meaningful engagement and help prevent CPSP in youth.

## 1. Background

Chronic postsurgical pain (CPSP) is defined as pain following a surgical procedure that persists for three months or longer and causes significant impairment in quality of life [1]. In pediatric populations, the prevalence of CPSP is estimated to be between 20% and 38% at 12-months post-surgery, with most youth reporting mild to moderate pain [2,3,4]. CPSP is increasingly prevalent, resulting in significant disruption to function, with some studies showing over 30% of youth experiencing some level of disability related to post-surgical pain beyond expected healing time [3].

There are several key physiological and psychological factors that place youth at greater risk of developing CPSP. Pain has a longstanding history as being a significant predictor of further pain [5]. In the surgical pain context, pain prior to surgery is a significant predictor of which youth will develop significant post-surgical pain or CPSP [6,7,8]. There are several known psychological risk factors, such as anxiety, mood disorders, and poorer coping with pain [9,10]. Caregiver anxiety and pain catastrophizing are also associated with CPSP [11,12]. To bring these factors together, a diathesis–stress model has been proposed as a critical lens through which to conceptualize the risk of developing CPSP [4,13], whereby one’s risk of developing CPSP increases with the number or severity of risk factors (e.g., anxiety sensitivity).

Given the multiple contributors to CPSP, a multidisciplinary approach to pain management is critical to support youth after surgery, whereby psychological assessment and psychological strategies are implemented alongside pharmacological and physical therapy [14]. Particular attention is especially warranted for psychological interventions given the role of psychological factors (e.g., anxiety and depressive symptoms) as risk factors for CPSP. Critical components of psychological interventions for postsurgical pain management include helping children and their caregivers understand how thoughts and feelings can modulate pain, as well as teaching cognitive behavioural strategies, distraction strategies, and relaxation strategies [14]. There is growing evidence to demonstrate that psychological interventions are associated with improvements in managing pain intensity in pediatric populations, as well as related psychological symptoms such as anxiety, emotional state, and fear [15,16,17]. Such interventions are also shown to be acceptable and satisfactory in their ability to teach youth strategies to manage psychological symptoms that arise in the context of surgical pain [18]. Psychological interventions for caregivers also show improvements in caregiver anxiety following their child’s surgery [16,19].

While there is promising evidence for the effectiveness of psychological interventions to manage postsurgical pain, there are inconsistencies in what is delivered within such interventions and knowledge gaps about how they work. In a recent systematic review on 14 psychological interventions for post-surgical pain management, the majority were single 30-min sessions delivered either prior to surgery or post-surgically, with only two interventions delivering content pre- and post-surgically [20]. Moreover, most psychological interventions in the literature predominantly focus on post-surgical pain, and there is a lack of evidence for interventions that focus on pain in the period prior to surgery through to several weeks following surgery. A validated and comprehensive approach to managing pain across the surgical timeline is necessary to ensure all components of pain management education and support are offered to optimize coping and pain management and reduce the risk of CPSP.

Beyond the content and structure of the available interventions that broadly address coping with surgery and related pain (i.e., interventions involving elements of psychological interventions, as well as other approaches like play therapy, preparation, etc.), the reviews conducted to evaluate their effectiveness are largely limited to quantitative methods (e.g., Fincher et al., 2012; He et al., 2015) [19,21]. While quantitative methods are essential to inform such evaluations, they are limited in their ability to elucidate the human experience of participating in an intervention. Indeed, the inclusion of qualitative data alongside quantitative data can illustrate the experience of partaking in an intervention and facilitate an exploration of why different components were effective or ineffective. The inclusion of qualitative data can also directly inform implementation plans for these interventions, as it provides an opportunity for participants to describe their experiences and perspectives on how the intervention should be adapted and used [22]. 

Evidence is building for the use of psychological and psychosocial interventions to mitigate the transition to CPSP; however, gaps remain in such approaches. To address these gaps, a psychological intervention was developed—termed the perioperative psychological program (PPP)—to (1) identify and target psychological risk factors for developing CPSP, and (2) improve self-management of pain throughout the operative timeline alongside youth and caregivers. The program takes a biopsychosocial approach to pain management and was developed in partnership with a multidisciplinary transitional pain team and a panel of patient partners to inform the content and delivery of this intervention (see Ruskin et al., 2024 [23] for more detail). Conducting this work specifically within the Canadian context not only allows for the consideration of unique factors within our healthcare system that may impact facets of pain (e.g., surgery wait times, universal healthcare covered procedures) but also lends itself well to consideration of how such an intervention may be used in similar healthcare climates (e.g., universal healthcare) where youth have access to multidisciplinary pain teams that include psychological services. The purpose of this mixed-methods study was to examine youths’ and caregivers’ satisfaction with, and acceptability of, the PPP program. This study also examined factors that supported youths’ and caregivers’ engagement with this intervention.

## 2. Methods

### 2.1. Design

The current study was conducted at a large urban pediatric tertiary care hospital and is part of a larger randomized controlled trial investigating the effectiveness of a psychological intervention for managing distress and pain across the surgical period. Ethical approval for this research was obtained from the institution’s Research Ethics Board (REB #1000075212). This study took a convergent mixed methods design, whereby quantitative and quantitative data were analyzed simultaneously and integrated to compare results and gain a comprehensive understanding of findings [24]. For the quantitative study component, a cross-sectional design was used to capture perspectives on acceptability and satisfaction following exposure to the intervention. For the qualitative study component, a qualitative description approach was used, which is recommended when looking to generate knowledge of factors impacting clinical experiences [25].

### 2.2. Intervention

The PPP was designed to: (1) improve patient and caregiver self-efficacy in managing and understanding biopsychosocial aspects of pain processing; and (2) target reduction of psychological risk factors for CPSP. The intervention consisted of four one-hour psychologist-led virtual sessions, three of which were delivered pre-surgically and one “booster” session delivered within two-weeks after surgery, in line with recommendations from Pagé et al., 2013 [11]. A psychology consult was provided at the first session to identify any specific surgical worries held by youth and to identify underlying risk factors for developing CPSP (e.g., anxiety, depressive symptoms, youth and/or caregiver pain catastrophizing). Session content included psychoeducation on the biopsychosocial nature of pain, teaching of psychological strategies to manage pain and psychological symptoms (e.g., pre-existing anxiety, mood, procedural distress), and the development of a coping plan for youth and families to manage pain and psychological symptoms during the peri-surgical period. Sessions were led by a clinical psychologist specialized in pain assessment and management (DR) and provided via virtual care (PHIPA [Personal Health Information Protection Act]-compliant Zoom for healthcare) (see Ruskin et al., 2024 for a detailed account of the intervention and its development) [23]. Standardized slide decks illustrating session content were created on Microsoft PowerPoint as part of the PPP intervention and utilized during each session. Slides contained hyperlinks that, for example, guided caregivers to sites on “helping your child prepare for surgery” and “what to expect on the surgery day,” while other hyperlinks took youth to audio recordings to practice relaxation and breathing exercises, and to a “comfort tool kit”. During each session, the therapist personalized the slides and information based on the youth’s responses (e.g., factors that open and close the “pain gates” along with an individualized coping plan completed jointly with the youth within the slide deck for the third session). Following each session, the slide deck was shared with the family so that youth could practice strategies between sessions and could access the information discussed, developed, and practiced at each session for future use. Patient partners were also engaged in the development of the intervention to identify relevant topics and resources to incorporate into the intervention (see Ruskin et al., 2024 for more details) [23].

### 2.3. Participants

**Figure 1:**
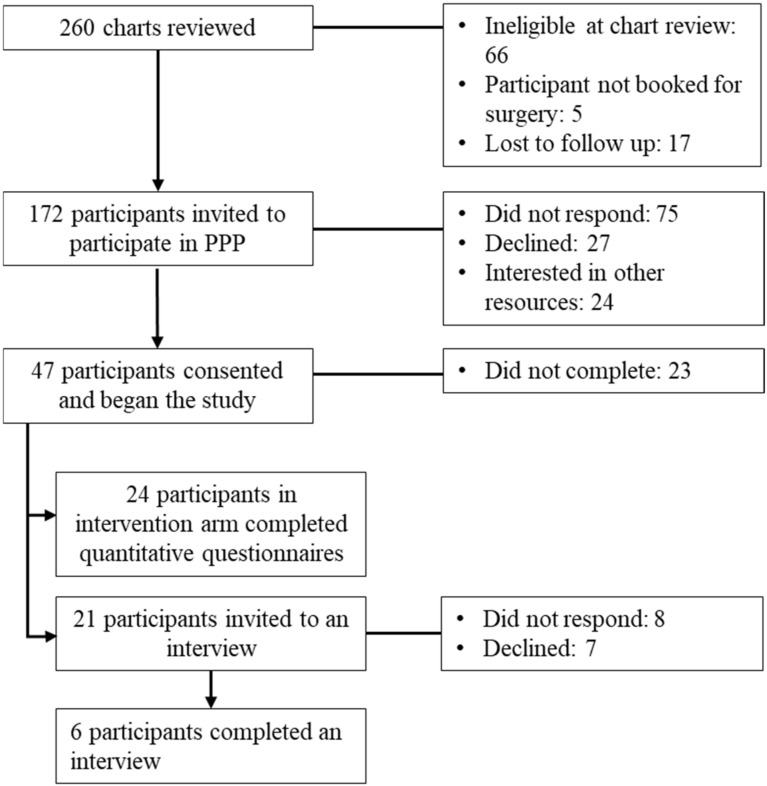
Recruitment flowchart.

Patients were eligible to participate in the current study if they were between 8 and 18 years old, proficient in English, and scheduled for surgery at the hospital where the study took place. Patients were ineligible to participate based on any of the following: inability to complete study forms because of either mental incapacity or language barrier; experiencing active psychosis or suicidal ideation requiring crisis services; diagnosed with cancer; received more than four sessions of outpatient psychological therapy for pain management in the six months prior to study screening; request from surgeon to assess suitability for surgery based on severity of acute and significant mental health challenges and referred for ongoing psychological treatment. A convenience sample of 260 patients scheduled for surgery and their caregivers were reviewed between November 2021 and September 2024. After chart review, 172 families were invited to participate and 47 provided consent. There were 24 participants enrolled in the intervention arm of the study and who are the focus of this paper. The other 23 participants were in the control arm of the study and received treatment as usual; thus, they were not included in the current paper as they did not receive the PPP intervention (See Figure 1—Recruitment Flowchart). The present sample size of 24 is aligned with the recommended sample size for feasibility research [26]. Of the 24 participants in the intervention arm of the study, 21 were invited to participate in interviews and 6 participated.

### 2.4. Measures

The primary outcomes of this study measured satisfaction with, and acceptability of, the PPP intervention. Treatment acceptability was measured by patient and caregiver satisfaction with the PPP via questionnaire and semi-structured interview. To characterize the study sample, demographics and reports of the following domains were collected at baseline: pain intensity, pain catastrophizing (youth and caregiver), and psychological outcomes (i.e., anxiety, depressive symptoms).

#### 2.4.1. Pain Intensity Numeric Rating Scale

This self-report measures pain intensity on a scale from zero (no pain) to ten (worst pain). Ratings included pain intensity most of the time over the past seven days as well as pain intensity at its worst within the past seven days. The Numeric Rating Scale has been shown to be both reliable (r = 0.96) and valid (r = 0.86 to 0.95) with chronic pain patients [27]. Youth completed this measure at baseline.

#### 2.4.2. Pain Catastrophizing Scale

The Pain Catastrophizing Scale (PCS) is a measure of the extent to which an individual catastrophizes about their experience of pain within the domains of rumination, magnification, and helplessness [28]. The PCS has been shown to have excellent internal consistency (α = 0.87) [28] and concurrent validity (*p* < 0.01) [29], as well as test–retest reliability [30]. The 13-item self-report questionnaire was administered to youth and caregivers at baseline.

#### 2.4.3. Patient-Reported Outcomes Measurement Information System (PROMIS) Measures

Functioning across multiple domains was assessed using the Patient-Reported Outcomes Measurement Information System (PROMIS) from the National Institutes of Health (NIH). Short form versions of the PROMIS measures were used for each domain [31,32] with items rated on a five-point ordinal scale from “Never” to “Almost Always.” All PROMIS measures used were the pediatric short form versions, which are shown to have excellent reliability above 0.85 [31,32,33] and are validated across various pediatric health samples [34].

**PROMIS Anxiety.** The PROMIS Anxiety is an 8-item scale that measures the presence of anxiety-related symptoms within the past seven days including fear, anxious misery, hyperarousal, and somatic symptoms.

**PROMIS Depressive Symptoms.** The PROMIS Depressive Symptoms is an 8-item scale that measures the presence of depression-related symptoms within the past seven days, including negative mood, views of self and social cognition, as well as decreased positive affect and engagement.

**PROMIS Pain Interference.** The PROMIS Pain Interference is an 8-item scale and measures the extent to which pain limits a child’s ability to engage with various activities (e.g., sleep).

#### 2.4.4. Patient and Caregiver Satisfaction Questionnaire

A satisfaction questionnaire was developed for this study to obtain feedback from participants in the intervention group regarding their experience with the PPP. Queried topics included program structure, intervention content, technical issues, and overall satisfaction. Response options varied across questions and included forced choice (yes/no), Likert Scale (e.g., from 1 [Completely Agree] to 8 [Completely Disagree]) and free text. Two versions of this questionnaire were developed for youth and caregivers, respectively. Each version explored the same topics with changes in wording to relate to the intended audience. An exploratory analysis of reliability was conducted with the 8-items specifically pertaining to satisfaction with intervention outcomes from the youth satisfaction questionnaire. These items demonstrated strong internal consistency (α = 0.98); however, this result should be interpreted with caution as it may not be reliable due to small sample size.

#### 2.4.5. Semi-Structured Interview

A semi-structured interview was administered to a subset of participants. The interview guide consisted of 13 questions pertaining to participants’ satisfaction and the acceptability of the intervention. The interview guide was designed to be administered to youth and their caregivers simultaneously. Interviews were audio recorded and transcribed verbatim.

### 2.5. Procedure

Following institutional REB approval, patients’ medical charts were reviewed for eligibility; those meeting eligibility criteria were introduced to the study by a member of their circle of care. Those interested were then approached by a member of the research team to obtain informed consent using an online consent form through Research Electronic Data Capture (REDCap), a secure, web-based application designed to support data capture for research studies [35]. Participants in the intervention group were scheduled for three 1-h virtual care sessions with DR delivered approximately weekly leading up to surgery and one “booster” session approximately 14 days after surgery, in addition to standard medical and transitional pain care. All PROMIS, pain intensity, and pain catastrophizing measures were administered at baseline. The satisfaction questionnaire and semi-structured interview were administered post-booster session.

### 2.6. Analytic Approach

#### 2.6.1. Qualitative Analyses

All semi-structured interviews were transcribed verbatim by a research volunteer (RR) and transcripts were then analyzed using inductive content analysis [36]. First, NEM (primary coder) and RR engaged in familiarization with the transcripts and a comprehensive code book was collaboratively generated inductively from the transcripts. Next, open coding was conducted using a line-by-line approach to apply codes to the data. To promote rigour and consistency in the coding approach, 50% of transcripts were double coded by NEM and RR, and minor differences were resolved [37]. The primary coder coded the remaining transcripts independently. Related codes were then grouped together into categories that described similar phenomena. Where there were distinct concepts within a larger category, subcategories were generated. Finally, categories were abstracted to generate descriptions of how the concepts with each category addressed the research question. To maintain rigour, an audit trail of resolutions and changes to the codebook was maintained and referenced in subsequent stages of analysis as necessary.

Open ended text responses were also analyzed with deductive content analysis as the guiding approach, whereby brief text responses to each question were coded line by line with codes generated from the data to represent responses to each question. Data interpretation was conceptualized within the categories generated in the earlier inductive analysis and relevant quotations were presented alongside those selected from the transcripts.

#### 2.6.2. Quantitative Analyses

All quantitative measures were analyzed using descriptive statistics (i.e., mean, standard deviation) and frequency analyses. Likert scale items administered as part of the satisfaction survey were collapsed into “agree” and “disagree” categories in the analysis phase.

#### 2.6.3. Mixed Methods Integration

In line with the convergent mixed methods approach, the quantitative and qualitative data were compared to explore consistency in the data and whether any further conclusions could be drawn through this comparison [24]. Qualitative data were analyzed first to ensure bias would not be introduced into the analysis based on quantitative findings. Quantitative data was summarized and compared to the findings of the qualitative data to determine consistency, discrepancy, and expansion of the data. These conclusions were then reviewed by the larger study team.

## 3. Results

### 3.1. Participants and Intervention Delivery Characteristics

A total of twenty-four participants completed the intervention and measures, six of whom took part in semi-structured interviews. The youth participants were primarily white females (58.3% female) with an average age of 14.1 years (see Table 1 for all demographics). The majority of participants had undergone spinal fusion or foot surgery, and most had not previously had surgery (54.2%) (see Table 1 for all surgical and pain history). Most participants identified having pre-existing pain (58.3%). Among participants who took part in an interview, the average time from the final PPP pre-surgery session to the surgery was 4.3 days (*SD* = 3.5 days). The average time from completion of the PPP pre-surgery sessions to the interview was 97.1 days, or approximately 14 weeks (*SD* = 19.6 days). The average time between surgery and the post-surgical booster session was 22.3 days, or approximately 3 weeks (*SD* = 5.8 days). The average time between the post-surgical booster session and the interview was 71 days, or approximately 10 weeks (*SD* = 21 days). The average time from surgery to the interview was 92.8 days, or approximately 13 weeks (*SD* = 18.1 days).

### 3.2. Qualitative Results

The inductive content analysis generated four categories: (1) developing support and a sense of preparedness for surgery; (2) connection matters between families and the team; (3) personal characteristics may impact intervention use; and (4) the need for adaptable content and delivery. Each category is described below and is accompanied by descriptive quotations from both the interview transcripts and relevant text from the open-ended response section of the satisfaction questionnaire.

#### 3.2.1. Category One: Developing Support and a Sense of Preparedness for Surgery

The youth and caregivers described that the PPP supported developing a sense of preparedness to manage procedural distress before surgery and pain related to their surgery. The specific skills taught within the context of the overall PPP approach was described as supporting youth and families to cope with the challenges that arose in the face of surgery, including pain, fear, and worry. There were two sub-categories generated within this overall category.

**Subcategory One: intervention components fostered a sense of preparedness.** The components of the PPP were helpful to families in supporting the development of a plan to manage pain and emotions across the surgical timeline, which supported them in feeling prepared for their upcoming surgery. Specifically, aiding in this sense of preparation prior to surgery was the perceived value of supporting materials (e.g., slides with pain coping plans) in providing clear guidance on how to manage pain and psychological symptoms before and after surgery, both independently and with caregiver support.

*“It was straight to the point; it was easy to read. Yeah. So definitely [youth] went through, and I went through it too.”*

(Caregiver of 14-year-old)

*“We’re able to still have the slides, it shows you what to review on our own.”*

(15-year-old youth)

While some families shared that they did not use the materials, they did share that the experience of selecting and reviewing strategies together was valuable in understanding how to manage pain.

*“We didn’t necessarily go back and reference it. But um building it together really helps because then it was kind of solidified and cemented for us.”*

(Caregiver of 14-year-old)

Youth and caregivers also described the PPP as helping them set expectations for what surgery and management afterward would be like. This was described as helping to manage anxiety and feel prepared for the procedure.

*“I think it’s important because as a parent, you want to know what the child is going [through], so it definitely prepares you. And as a child, it gives you the information in regards to surgery…”*

(Caregiver of a 14-year-old)

*“[The intervention] helped me understand what I was walking into, properly preparing me for the upcoming surgery and when the date came, I felt much more comfortable than I would if I hadn’t gone to the sessions.”*

[Open-text response from 16-year-old Youth]

While some youth reported learning new strategies through the PPP, others described repetition or overlap with known pain and anxiety management strategies. For many youth, this was a positive feature of the intervention and reinforced and refreshed knowledge of how to manage these experiences.

*“…Some of my information did [overlap], but I think it’s good to hear twice though, like, even if it was because some of them…I forget some things and then see it again. And then I heard [the therapist] before like, but I think I, it wasn’t repetitive or anything. …[The therapist] was telling me good stuff. Like, all good information I could use.”*

(15-year-old youth)

*“I liked that the sessions reinforced things we already knew and gave us new strategies. It allowed my child to be reminded of the strategies.”*

(Open-text response from a Caregiver of a 14-year-old)

**Subcategory Two: outcomes of Engaging with the PPP Intervention.** Youth and families described implementing the strategies and resources learned through the PPP and highlighted helpful outcomes in terms of managing pain and emotions after surgery.

*“I found that helpful [at the] beginning so that I was prepared with things that I could use and do to try and take her mind off the pain and stuff.”*

(Caregiver of 16-year-old)

Many families used specific and preferred strategies to manage pain after the youth’s surgery.

*“I find the most helpful five, four, three, two, one. And um distractions.”*

(14-year-old youth)

*“…[The therapist] did um two things that I found were really helpful. They were both just kind of, like, closed my eyes and just listened to [the therapist’s] voice. And [the therapist] said, oh, just like, calm your toes down, and then [the therapist] went all the way up, and then it was just like finding your happy place. I found those were incredibly helpful.”*

(16-year-old youth)

Some caregivers specifically described the value of different strategies depending on the severity of pain the youth was experiencing.

*“In regards to the different pain levels and things that we could do to help [youth], especially when she was after the surgery. That was really helpful.”*

(Caregiver of a 14-year-old)

*“Distraction is a big thing for her. You get her talking about something that she likes, and then all of a sudden, pain. She talked for 20 min, still being in pain, but at least sidetracked those moments.”*

(Caregiver of a 16-year-old)

In addition to pain, many youth and caregivers identified the value of the PPP in providing strategies for managing psychological symptoms associated with their surgery, such as anxiety, fear, and frustration, among other challenging experiences, such as changes in mobility.

*“What we did find super, super helpful is not only pain, but we’ve been using them for frustration levels and coping with the immobility that we have. Because [youth] still has not been walking. And it’s very hard at 14 to um lose your independence. And so, most of our biggest challenges had been around that frustration. And we’ve actually used the coping strategies, so so much around that.”*

(Caregiver of 14-year-old youth)

Youth and caregivers’ impressions of the PPP were positive overall in terms of the benefit and outcomes of participating in the sessions. Families reported that they would recommend the PPP to other youth undergoing surgery and their caregivers seeking support for their post-surgical transition.

*“I would. I would recommend it to those people who feel like they need it. Because some people might not need the same things everybody else needs.”*

(13-year-old youth)

*“I would definitely recommend the program to other parents and patients, letting them know what to expect. Um. Especially for like if they have a child who’s had pain for a long time, like my child.”*

(Caregiver of a 14-year-old)

Overall, through resources that provided youth and caregiver with strategies to manage pain and other psychological symptoms, as well as reinforcing known strategies, youth and caregivers found that the PPP provided valuable preparation for surgery and pain management.

#### 3.2.2. Category Two: Connection Matters Between Families and the Team

The importance of interpersonal connections was also highlighted by youth and caregivers who participated in the PPP. The opportunity to have needs met in a way that was comprehensive and dynamic was an important element of the approach.

Caregiver involvement was an integral part of the intervention and a key connection between children and their caregivers fostered by the PPP. Many youth described a preference for having a caregiver present during sessions to support their engagement in, and use of, the PPP strategies to manage pain and worry prior to and following surgery.

*“I think when I was really stressed, I would just be able to lean on my parents and help them talk me out of bad situations.”*

(16-year-old youth)

*“I liked being with my mom constantly. I liked it. Plus, the surgery is more… it’s affecting the both of us anyway, so both need to be there because she has 8 h to wait in a waiting room, so might as well just sit in and learn some skills.”*

(14-year-old youth)

Caregivers themselves also expressed satisfaction with their involvement in the PPP, while some wished to be involved even further. Many described the benefits of being familiar with the approaches taught in the sessions and how they can be used, which was seen as promoting their ability to support their child post-surgically.

*“[The therapist] sometimes talked to me for a couple of minutes in the beginning or both of us in the beginning, and then talk to [youth] and then kind of do a little recap at the end with me to see if I had any questions or if [the therapist] had questions for me or whatever or if there was something that [youth] maybe wanted to share with me but couldn’t. I like the way that was set up.”*

(Caregiver of a 16-year-old)

*“I found that as parents we were unsure of the advice. Some of the calls excluded the parents—which we understand—but we felt we didn’t have a complete understanding of what was happening.”*

(Open-text response from the caregiver of a 15-year-old)

In another instance, connection facilitated comfort, especially among one youth who had social anxiety. The opportunity to involve the caregiver in the intervention helped the family gain access to information even when youth themselves may have struggled to engage.

*“[Youth] has really bad social anxieties, like, all the social anxiety. So sometimes doing these meetings, that’s one of the reasons why she didn’t want to do this one is it’s a lot for her. So they were all really hard for her to do initially.”*

(Caregiver of 16-year-old youth)

Another key role for connection that the PPP facilitated was between the therapist and the youth. Within their positive feedback regarding the psychologist, the youth specifically commented on the value of the psychologist taking the time to understand the youth’s fears and questions. The youth felt particularly validated through the opportunity to ask questions and gain information specific to their needs.

*“I like how it was like there was a certain like, like it wasn’t…rushed. …[The therapist] was taking her time to explain and like make sure that I’m understanding and all that like it wasn’t like brushed off or something.”*

(15-year-old youth)

*“It felt really helpful at the time. It really did. I like talking to [the therapist]. … I don’t remember it all, but I know it was like, oh, I’m glad [the therapist] told me that, or, I’m glad [the therapist] mentioned that…”*

(16-year-old youth)

The connections fostered by this intervention extended beyond those immediately participating in the intervention to include other members of the medical team. Caregivers valued the opportunity to share information needs with the psychologist who could advise where to direct questions or the opportunity to get help from the pain team with directing information to relevant health professionals. Some families described how this offered connections with other professionals they had not previously known about but benefitted greatly from.

*“I think that um it was super helpful for [youth] to talk about some of the things she was worried about, because [Therapist] was really great at letting us know who we should tell, who it was important to let know. And then [the therapist] also set up like child life specialists for us and some other things like that, that we wouldn’t necessarily have known um that we were able to access.”*

(Caregiver of 14-year-old youth)

*“…[Therapist] gave info to the other departments about [youth’s] specific worries and helped me know which departments dealt with what issues.”*

(Open-text response from the caregiver of a 14-year-old)

The intervention overall created a sense of connection not only among youth and caregivers, but between families and their care teams. Creating a sense of comfort, characterized by open communication and understanding, were key characteristics that supported families’ engagement with the PPP intervention.

#### 3.2.3. Category Three: Personal Characteristics May Impact Intervention Use

While there were many helpful characteristics and outcomes of the PPP, some youth and caregivers did not connect as readily with the intervention, or experienced challenges implementing the recommendations. Among the few who experienced such challenges, most were related to patient and family needs, preferences, and characteristics.

Some challenges with using the strategies and resources in practice related to the youth and family’s individual level of readiness to engage with the resources, with barriers ranging from remembering to use the intervention to willingness to use the strategies. Indeed, for some families, readiness appeared to be impacted by forgetting to use the strategies or slides they had been provided, and some youth may have benefitted from further caregiver support to remember to implement them.

*“I remember being sent [the slides]. But I always forgot to get them…when I had my surgery. It would have been easier if you just gave like a printed copy to the parents…”*

(13-year-old youth)

*“It’s still really hard for her to remember in the moment to pull [the strategies] out. But if we remind her, then she knows the skills and she knows the tools.”*

(Caregiver of a 14-year-old)

For other families, however, readiness was hindered by resistance to using these strategies, in some cases due to hesitation to try them or a belief that they would not provide support.

*“But [the medical team] also really encouraged her to try the [strategies] that she thought wouldn’t work. …she just didn’t want to try other things. And when she’s in pain, she’ll tell you she doesn’t want to use the distraction either.”*

(Caregiver of a 16-year-old)

*“My child is very resistant to new ideas and she doesn’t believe any will work.”*

(Caregiver of a 16-year-old)

A subset of families experienced specific challenges engaging with the PPP seemingly brought about by expectations and experiences of pain. One family described that preemptively hearing about pain through the intervention, and prior to surgery, caused significant fear and distress.

*“I didn’t realize how much hearing the term pain management over and over again back in November and December for myself was very triggering. [Child] was not in pain, so she never had pain.”*

(Caregiver of a 16-year-old)

Conversely, a different family described that despite the focus on and preparation for pain afforded by the PPP, the pain they experienced significantly exceeded their expectations.

*“I didn’t have a whole lot of fears or worries going into [the surgery]. We knew for a long time it was coming. My fears and anxieties more came when I first saw her after the surgery and saw how much pain she was in. I don’t think anything could have really prepared me for the amount of pain that she was going to get.”*

(Caregiver of a 16-year-old)

Among families who experienced challenges implementing the strategies and resources provided by the PPP, many of these challenges appeared related to individual characteristics and concerns.

#### 3.2.4. Theme Four: The Need for Adaptable Content and Delivery

Adaptability of session content was highlighted as a key facet of ongoing PPP development. There were positive components of the intervention as delivered in its current form; however, opportunities were identified to create further flexibility in both the content delivered, and how it is offered to youth and caregivers.

**Subcategory One: opportunities for adapting PPP content.** Youth and families identified areas where potential adjustments to the PPP could be made to increase its relevance and scope to better align with the needs they experienced throughout their surgical journeys. For example, one teen indicated they would have preferred intervention content to be specific to the surgery they underwent.

*“I think, too, though, it was for general pain, it wasn’t specifically related to my surgery. So … it was…I know it’s really hard to be really specific, to have one person for everything, but … that would have been more helpful.”*

(16-year-old youth)

A caregiver noted that some strategies should be adjusted based on the patient’s age.

*“A couple of them that were um maybe not they were maybe geared to younger kids instead of older kids.”*

(Caregiver of a 14-year-old)

**Subcategory Two: opportunities for integrating flexibility in the PPP delivery.** Youth and families offered perspective on ways to both adapt the PPP structure as well as integrate greater flexibility in how the intervention was delivered based on family preferences. While the majority of participants indicated the structure of the intervention worked well, there were some suggestions. For example, one teen shared that waiting two weeks to receive a booster session following surgery was difficult.

*“…Because it was like it was just right after the surgery, you would want some type of like, “oh, like, what am I supposed to feel this way, this way?” Because after you’re healing on your own, like, you’re just like, like, you’re doing by yourself, if you’re just waiting two weeks, like that two weeks is like really crucial, you know? So it’s like, I’m by myself for those two weeks. It would be better is they were here, like the week right after.”*

(Caregiver and 15-year-old youth)

Another youth shared that they preferred sessions continue to be delivered in a one-on-one fashion, which they expressed facilitated a sense of safety.

*“Um I feel like the group one could be good for other people, but I feel like one on one would have been better for me. Um, with other people around I don’t feel as safe as if there was just one person.”*

(13-year-old youth)

Alternatively, another youth preferred to have an opportunity to learn from and connect with similar age peers through this intervention process.

*“I wanted someone in my own age and not somebody that’s going to try to analyze me and my feelings is somebody to feel emotions with.”*

(16-year-old youth)

Finally, other participants suggested being mindful of all the other appointments occurring right before surgery and felt that it was a challenging time to fit in sessions.

*“It was a lot of sessions in a very busy time with other pre-op appointments.”*

(14-year-old youth)

### 3.3. Quantitative Results

#### 3.3.1. Baseline Characteristics

Youth and caregivers completed measures of psychological symptoms at baseline (Table 2). Among all 24 participants, mean scores on mental health questionnaires (i.e., depression, anxiety) and youth pain catastrophizing were within normal ranges; however, parent pain catastrophizing was in the clinically significant range *(M* = 61.07, *SD* = 10.99). On average, the youth’s baseline pain intensity was mild (*M* = 3.54 *SD* = 2.66), while their worst pain was in the moderate range (*M* = 4.63, *SD* = 3.19). For youth who participated in the interviews (*n* = 6), mean pain catastrophizing, anxiety, and pain interference reached the clinically significant range, while caregiver pain catastrophizing ratings were in the average range. The youth’s ratings of baseline pain intensity indicated mild overall pain (*M =* 3.83, *SD =* 2.86), while the worst pain was in the moderate range (*M =* 5.17, *SD =* 3.37).

#### 3.3.2. Youth Satisfaction with the PPP Intervention

Youth who participated in all sessions of the PPP intervention (*n* = 24) completed a satisfaction questionnaire. As reported in Table 3, the youth reported satisfaction with the number and timing of sessions pre- and post-surgery, their caregivers’ involvement in sessions, and the individual format. The majority of youth also preferred the virtual delivery of the sessions. Most youth preferred to have the sessions occur within two weeks prior to their surgery. As reported in Table 4, most youth agreed that the PPP provided information and new strategies to manage worries and pain related to surgery, with the most popular strategy being physical pain management. The majority of youth agreed that the personalized pain coping plan was helpful. As reported in Table 5, nearly all youth agreed that the PPP helped them manage worries about surgery and prepared them for the surgery. Most youth agreed that the PPP prepared them to manage pain, improved their pain-coping skills, and improved confidence in managing post-surgical pain. The majority reported receiving support from the therapist regarding other challenging areas (e.g., anxiety). Nearly all agreed that they would recommend the program to other youth.

#### 3.3.3. Caregiver Satisfaction with the PPP Intervention

Caregivers (*n* = 24) who participated in all the PPP sessions reported satisfaction highly similar to that of youth. As reported in Table 3, the majority of caregivers were satisfied with the number and timing of sessions before and after surgery. They also preferred to have sessions occur within two weeks prior to surgery. Caregivers felt their involvement in the sessions overall was helpful and appropriate. They preferred one-on-one sessions over other formats, such as groups or workshops with other families. Most caregivers preferred virtual delivery and reported comfort in virtually interacting with the therapist. Caregivers predominantly reported the PPP gave both themselves and their child enough information to cope with pain and worry related to surgery, as reported in Table 6. The majority agreed that the personalized coping plan was helpful and that they would recommend the program to other families.

### 3.4. Data Integration

The quantitative and qualitative results were compared to determine whether data converged (i.e., was consistent), diverged (i.e., conflicted), or provided elaboration (i.e., one type of data provided additional information about a concept). Generally, there was convergence across the data. Participants consistently reported satisfaction with caregiver involvement, strategies, resources, preparedness, and whether they would recommend the program. There were some instances where qualitative data elaborated on the quantitative. Participants’ preference for timing being closer to surgery was elaborated on by highlighting benefits and drawbacks of session timing options (i.e., closer dates promoted the relevance of session material, while earlier dates allowed for more time for scheduling). While findings indicate youth primarily preferred one-on-one delivery of the PPP, qualitative data also highlighted the desire for peer connection through groups. Youth and caregivers also elaborated on the desire for more specific information about setting pain expectations and managing anxiety. One area where divergence emerged was in terms of the coping plan, where satisfaction was variable in the qualitative data, although it was rated positively in the quantitative data. Specifically, many participants shared that they had forgotten about the coping plan, but among those who did use it, it was seen as a benefit. Therefore, the positive quantitative data may reflect the positive perspectives of those who used the coping plan.

## 4. Discussion

The present study explored the satisfaction with, and acceptability of, the Perioperative Pain Program, a multi-session psychological intervention delivered pre- and post-surgery for the prevention of CPSP in youth. Results indicated satisfaction with the PPP, characterized by a sense of preparedness for surgery and pain management through learning strategies to cope with pain and psychological symptoms prior to and following surgery, especially at the critical 2-week post-surgical mark [11]. It also highlighted the benefits afforded through connections fostered with team members. Opportunities for further refinement include tailoring of the program based on surgery type and offering flexibility in delivery. This program was viewed as acceptable, aligning with outcomes of similar work in post-surgical care demonstrating that psychological intervention across the perioperative timeline is appropriate and acceptable [18]. Considerations for implementation in clinical contexts, as well as potential adaptations to this program, are discussed in further detail below.

### 4.1. Tailoring the Intervention’s Focus

Adaptations to the PPP content were among the most prevalent adjustments suggested. For example, two families raised concerns around how much information they received about pain. While one family described not receiving enough information about pain after surgery, another felt overwhelmed by the information about pain, which increased their anxiety. While setting expectations about pain is a key component of psychoeducation within such interventions, it may be appropriate to tailor the extent to which pain is discussed based on a youth’s preference. One strategy for addressing this could be adjusting the terminology to reduce pain language and increase positive coping language (e.g., “gate control theory of pain” could be rephrased as the “gate control theory of coping”). Indeed, providing youth with a sense of control around psychosocial elements of pain management, such as language and choice about what they would like information about, may in turn support pain management [38,39]. Moreover, such adaptations could promote “approach coping,” where fear and avoidance are moderated with support and cognitive–behavioural strategies [40]. Providing strategies that avoid overemphasizing pain yet are solution-focused may be a key opportunity to tailor interventions while still teaching strategies that can be generalized to coping and pain as needed.

### 4.2. Group Versus Individual Intervention

Many of the youth who participated in this intervention valued the opportunity to connect individually with the therapist; however, some of the youth were interested in a group-based delivery of the intervention to build connections with similar-age peers, which the adjacent literature on adults and youth with chronic pain show benefits for in terms of mobility and pain management. For example, in adults undergoing spinal fusion surgery, those who participated in a cognitive behavioural therapy group reported increased mobility after surgery and lower analgesic intake, relative to controls [41]. Benefits are also shown for caregivers, who in a caregiver-targeted group for youth chronic pain decreased unhelpful pain-related behaviours [42]. The opportunity to have peer support may also be an important element of the group therapy experience. Indeed, research in youth with chronic pain has shown peer support to increase coping and self-management of pain [43]. Group-delivered interventions also have financial benefits, given that multiple youth can be supported at one time, making this style of intervention more cost efficient [44].

Individually delivered interventions have shown benefits for teaching pain management and anxiety coping strategies for pre-surgical anxiety and post-surgical pain [45,46]. While there are no studies that compare individual and group delivered interventions to address post-surgical pain, a comparison of group versus individually delivered psychological interventions in adolescent chronic pain showed no significant differences in outcomes [47]. The ability to tailor content to youth, however, is a direct benefit of individual delivery, where therapists may address specific questions with relevance to the upcoming procedure. Moreover, anxiety is often a significant barrier to participating in groups [48]; thus, individual delivery may provide a more approachable entry point to therapeutic support for pain management. Practically speaking, individual delivery eliminates logistical challenges of coordinating multiple youth with a wide range of surgery dates that are subject to change. This may result in youth missing sessions, a shortcoming in all group interventions resulting in missed content [49]. Individual delivery can be an optimal approach to meet the unique needs of youth, mitigating the decay of recall and interruptions to care, while capitalizing on motivation to learn skills as the surgery date approaches.

There are benefits and drawbacks to delivering psychological interventions for pain via individual and group modalities. Contextual factors and patient needs should remain at the forefront when deciding how to deliver care. To support youth who wish to have group support, future work may consider comparing outcomes and satisfaction with group versus individually delivered sessions.

### 4.3. Severity of Mental Health in the Present Sample

The PPP intervention was originally intended to target youth with significant mental health risk factors for developing CPSP (i.e., high levels of anxiety, depression, pain catastrophizing). However, due to recruitment challenges posed by COVID-19 (e.g., significant slowdowns or complete stoppage of non-urgent surgical cases), eligibility criteria for the study were widened to include all youth seen by the transitional pain service for an upcoming surgery. As a possible consequence, participants generally presented with less severe mental health (i.e., average to just above average scores on mental health measures). While the intervention was found to be acceptable and satisfactory within this sample, it is possible that the acceptability and value of the intervention may have differed in a sample with a more severe mental health profile. For example, the literature on youth anxiety and CPSP indicate that trait anxiety is positively associated with CPSP, and when youth undergoing surgery receive cognitive behavioural intervention for pain, their anxiety one year later is significantly lower than those who do not receive intervention [50,51]. Thus, a sample with a more severe anxiety and mood profile may have had a greater need for strategies to manage mood and anxiety, and may have preferred earlier intervention to allow more time to learn strategies to manage mood and anxiety symptoms. As this work progresses, continuing to explore outcomes in the context of mental health characteristics may help inform whether psychological severity should be screened for and influence triage into a program such as the PPP. This could help ensure that the youth in most need of these services are supported, while conserving financial services implicated in delivering these types of services.

### 4.4. Caregiver Support as a Key Component of the Intervention

Youth and caregivers alike described caregiver involvement in the PPP as acceptable and beneficial. Youth described caregivers as key supports for using PPP strategies, while caregivers reaped benefits from learning about strategies to manage their child’s pain and anxiety. The benefits of caregiver involvement in the present work aligns with a growing literature that shows caregiver engagement to be a critical component of pain management for youth, through improvements in caregivers’ supportive behaviours and related improvements in youth psychological factors, such as readiness to change and functional disability [42,52]. Given the importance of caregiver involvement in this intervention, future iterations should continue to integrate this element.

### 4.5. Strengths and Limitations

This study has a number of strengths that support the integrity of its findings. The mixed methods design is a significant methodological strength of this study, adding detail to the quantitative findings through the nuance presented in the quotations, which can support the implementation of the findings. Moreover, the analysis of open-text responses in the satisfaction questionnaire data added evidence to illustrate the acceptability of the intervention from this supplementary lens. There were also a range of surgical and pain experiences captured within the sample of participants. This strengthens the transferability of the study results as conclusions are based on a broader surgical and pain experience, which may be more representative of the breadth of needs experienced by youth. Finally, the inclusion of caregivers as a participant group was a major strength as it ensures the recommendations made in this study reflect the needs of this group, which can promote more effective pain management for youth.

This study was not without its limitations. Firstly, sampling bias may have influenced the participants in the sample, and results may reflect a particularly motivated subset of the population, potentially limiting the generalizability of these findings. Attrition may have also contributed to this bias. Indeed, attrition rates are a common challenge in child health and behaviour change research, typically near 20% [53,54]; however, additional factors such as surgery dates and competing priorities during planning for surgery may have elevated the present attrition rates. Indeed, these results may therefore be limited to describing the experience of more motivated individuals who wish to engage with such an intervention and may be less representative of the experiences of patients who are less motivated. Additionally, the average time from booster session to interview was approximately 10 weeks. This time span occurred due to challenges scheduling participants in a timely way after their procedure (the interview was intended to occur six weeks post-surgery). While this timeline this may have hampered recall of details regarding their surgical experience or the intervention, it is balanced with the need to ensure adequate time between exposure and evaluation for the participant to utilize the intervention [55]. Finally, the qualitative data presented in this study only includes six participants out of the total sample of twenty-four. The total number of interviewees aligns with qualitative practices in health psychology [56] and while sample size does not threaten the validity of results in qualitative research, it is possible that the overall results may have been different had additional participants taken part. When interpreting qualitative research, the transferability of results should be considered in the context of factors that exist within one’s own setting.

### 4.6. Future Research Directions

The present research demonstrates that the PPP intervention is both acceptable and feasible to use among youth and their caregivers. Given the prevalence of chronic pain following surgical intervention, this research serves to benefit future youth who are at risk of developing CPSP. More specifically, it will ensure that interventions intended to provide psychological care are relevant to their needs and delivered in a way that meets these needs. Youth and caregivers who have access to such interventions through multidisciplinary pain teams will ideally benefit from this fine-tuned approach to not only teaching pain management skills, but addressing specific questions, leaving families feeling prepared for and supported to manage pain post-surgery. Future research should continue to explore other key indicators of success with this particular intervention and others like it, such as through examining the clinical effectiveness on post-surgical pain management.

In terms of care delivery, and given that health care dollars are limited, this interventions’ model of one-to-one delivery of the intervention may not be feasible for most institutions. Thus, future research can assess whether delivering the intervention content in a group format has similar outcomes to individual delivery.

Finally, many children in the present study were unable to access the PPP intervention because they were referred to the intervention too late (i.e., their surgery date was within a few days of the referral). Thus, future research should focus on evaluating solutions that encourage earlier referrals so that youth have the opportunity to have consults that identify risk factors for development of CPSP prior to surgery, while also promoting sufficient runway prior to surgery to address these risk factors (e.g., pain catastrophizing, anxiety/mood issues) prior to receiving surgery.

## 5. Conclusions

Psychological interventions are integral to the comprehensive approach to preventing CPSP. Current interventions in this area are primarily brief and educational in nature, whereas the PPP identifies specific risk factors and worries related to the upcoming procedure and addresses them through teaching psychological coping skills. This study contributes important evidence indicating that a more fulsome psychological intervention is acceptable and satisfactory among youth and caregivers in supporting their ability to cope with pain and anxiety, both prior to and after surgery. Taking a mixed-methods approach to this work provided rich detail on the personal experience of this intervention by including personal perspectives alongside rating data. It also highlights key considerations for successful implementation, including building connections among families and clinicians, adapting programming to meet individual needs through timing and delivery of the program, and offering tailoring considerations to proactively mitigate the development of CPSP in youth.

## Figures and Tables

**Table 1:** Youth participant demographics.

Variable	*n* (%)	*M* (*SD*)	Min	Max
Assigned Sex				
Female	14 (58.3)			
Male	9 (37.5)			
Missing	1 (4.2)			
Age (Years)		14.1 (2.1)	9	18
Ethnicity				
White	14 (58.3)			
Black	4 (16.7)			
South Asian	3 (12.5)			
East Asian	1 (4.2)			
Prefer not to answer	2 (8.3)			
Surgery Type				
Scoliosis	9 (37.5)			
Foot	9 (37.5)			
Pectus	4 (16.7)			
Cranioplasty	1 (4.2)			
Other (“ortho”)	1 (4.2)			
History of Pain				
Yes	14 (58.3)			
Duration of pain (years)		5.2 (4.5)	0.1	16
No	7 (29.2)			
Past Surgery				
No	13 (54.2)			
Yes	10 (41.2)			
One	7 (70.0)			
More than one	3 (30.0)			
Years since last surgery		5 (3.7)	1	12
1–3	4 (40.0)			
4–6	3 (30.0)			
7–9	1 (10.0)			
10+	2 (20.0)			

Note. *n* = 24; *M* = mean; *SD* = standard deviation; min = minimum value in the range; max = maximum value in the range.

**Table 2:** Youth and caregiver outcome measures at baseline.

Measure	Mean Value (*SD*)	Range
All study participants (*n* = 24)		
Youth Pain Catastrophizing Scale (*t* score)	57.42 (12.72)	30.33–88.18
Parent Pain Catastrophizing Scale (*t* score)	61.07 (10.99)	35.18–77.61
Pain Intensity—overall (mean)	3.54 (2.66)	0–8
Pain Intensity—worst (mean)	4.63 (3.19)	0–9
PROMIS Anxiety (t score)	54.37 (12.28)	33.5–74.6
PROMIS Depression (*t* score)	51.92 (12.77)	35.2–71.4
PROMIS Pain Interference (*t* score)	58.51 (10.99)	34–75
Interview participants (*n* = 6)		
Youth Pain Catastrophizing Scale (*t* score)	61.00 (14.08)	41.90–88.18
Parent Pain Catastrophizing Scale (*t* score)	57.40 (9.18)	47.30–72.56
Pain Intensity—overall (mean)	3.83 (2.86)	0–7
Pain Intensity—worst (mean)	5.17 (3.37)	0–8
PROMIS Anxiety (*t* score)	61.15 (11.51)	44.9–74.6
PROMIS Depression (*t* score)	57.33 (12.57)	35.2–71.4
PROMIS Pain Interference (*t* score)	61.18 (6.92)	51.7–71.5

Note. All study participants total is inclusive of interview participants.

**Table 3:** Youth and caregiver feedback on format and modality.

Outcome	Youth	Caregiver
	*n* (%)	*n* (%)
The timing of the PPP sessions relative to the surgery were:		
Just right	18 (75.0)	21 (87.5)
Too close to surgery date to be helpful	2 (8.33)	2 (8.3)
Too far away from surgery date to be helpful	4 (16.7)	1 (4.2)
If it were my choice, the three sessions before the surgery should occur:		
2 weeks before surgery	11 (45.8)	9 (37.5)
Less than 2 weeks before surgery	9 (37.5)	7 (29.2)
1 month before surgery	3 (12.5)	7 (29.2)
3 months before	0 (0)	1 (4.2)
Did not respond	1 (4.2)	0 (0)
The number of the PPP sessions before the surgery was:		
Just right	19 (79.2)	21 (87.5)
Too many	3 (12.5)	2 (8.3)
Too few	1 (4.2)	1 (4.2)
Did not respond	1 (4.2)	0 (0)
The number of the PPP sessions after the surgery was:		
Just right	21 (87.5)	18 (75.0)
Too few	3 (12.5)	6 (25.0)
I would have preferred if the PPP sessions were offered in a group format with other teens who are going to have surgery.		
Disagree	18 (75.0)	1 (4.2)
Agree	6 (25.0)	23 (95.8)
I feel that having a caregiver involved in the PPP was helpful.		
Agree	21 (87.5)	23 (95.8)
Disagree	3 (12.5)	1 (4.2)
The amount of caregiver involvement in the PPP sessions was:		
Just right	22 (91.7)	18 (75.0)
Too much	1 (4.2)	0 (0)
Too little	1 (4.2)	5 (20.8)
Did not respond	0 (0)	1 (4.2)
Modality Preference		
Virtual by video conference	20 (83.3)	22 (91.7)
In person	4 (16.7)	2 (8.3)
I felt comfortable interacting with my healthcare providers over video.		
Agree	23 (95.8)	24 (100.0
Did not respond	1 (4.2)	0 (0)

Note. *n* = 24.

**Table 4:** Youth satisfaction with strategies and content.

Outcome	*n* (%)
My personalized coping plan provided at the end of the PPP was helpful.	
Agree	22 (91.7)
Disagree	1 (4.2)
Did not respond	1 (4.2)
The PPP sessions provided me with helpful information and strategies to help me learn different ways to cope with my pain and any worries related to the surgical experience.	
Agree	21 (87.5)
Disagree	2 (8.3)
Did not respond	1 (4.2)
Overall, the PPP taught me new strategies and skills for managing pain.	
Agree	20 (83.3)
Disagree	3 (12.5)
Did not respond	1 (4.2)
Skills considered helpful:	
Breathing techniques	19 (79.2)
Understanding how pain works in the body	20 (83.3)
Relaxation practices	21 (87.5)
Anxiety reduction	21 (87.5)
Challenging negative thoughts	15 (62.5)
Self-hypnosis	13 (54.2)
Physical pain management	22 (91.7)
Strategies to reduce fears associated with surgery	20 (83.3)
Personalized coping plan	19 (79.2)

Note. *n* = 24.

**Table 5:** Youth satisfaction with outcomes.

Outcome	*n* (%)
The PPP helped my worries about the surgical experience.	
Agree	22 (91.7)
Disagree	1 (4.2)
Did not respond	1 (4.2)
The PPP improved my ability to cope with pain.	
Agree	20 (83.3)
Disagree	3 (12.5)
Did not respond	1 (4.2)
The PPP prepared me on how to manage my pain.	
Agree	23 (95.8)
Did not respond	1 (4.2)
The PPP improved my confidence in managing pain after the surgery.	
Agree	21 (87.5)
Disagree	2 (8.3)
Did not respond	1 (4.2)
Overall, the PPP was helpful in preparing me for my surgical experience.	
Agree	21 (87.5)
Disagree	2 (8.3)
Did not respond	1 (4.2)
Did your therapist providing the PPP help identify any other challenging issues for you (e.g., anxiety, low mood) that needed to be addressed in addition to the surgical pain?	
Yes	14 (58.3)
No	9 (37.5)
Did not respond	1 (4.2)
Overall, I would recommend the PPP for other children undergoing surgery and their families.	
Agree	22 (91.7)
Disagree	1 (4.2)
Did not respond	1 (4.2)

Note. *n* = 24.

**Table 6:** Caregiver satisfaction with content and outcomes.

Outcome	*n* (%)
My child’s personalized coping plan provided at the end of the PPP was helpful.	
Agree	21 (87.5)
Disagree	3 (12.5)
The PPP sessions provided my child with enough information to have different ways to help them cope with their pain and worries related to the surgical experience.	
Agree	22 (91.7)
Disagree	2 (8.3)
The PPP sessions provided me with enough information to have different ways to help my child cope with their pain and worries related to the surgical experience.	
Agree	22 (91.7)
Disagree	2 (8.3)
Skills considered effective for child:	
Worries about the surgical experience	21 (87.5)
Ability to cope with pain	17 (70.8)
Preparing to manage pain	18 (75.0)
Improving confidence in managing pain	17 (70.8)
Improving day to day function	10 (41.7)
Teaching new pain management skills and strategies	17 (70.8)
Skills considered effective for caregiver:	
Worries about the surgical experience	18 (75.0)
Ability to help child cope with pain	18 (75.0)
Preparing to help child manage pain	18 (75.0)
Improving confidence in managing child’s pain	15 (62.5)
Teaching new pain management skills and strategies	15 (62.5)
Overall, I would recommend the PPP for other children undergoing surgery and their families.	
Agree	23 (95.8)
Disagree	1 (4.2)

Note. *n* = 24.

## Data Availability

The data presented in this study are available on request from the corresponding author due to the nature of the data.
